# Automated volumetric evaluation of intracranial compartments and cerebrospinal fluid distribution on emergency trauma head CT scans to quantify mass effect

**DOI:** 10.3389/fnins.2024.1341734

**Published:** 2024-02-19

**Authors:** Tomasz Puzio, Katarzyna Matera, Karol Wiśniewski, Milena Grobelna, Sora Wanibuchi, Dariusz J. Jaskólski, Ernest J. Bobeff

**Affiliations:** ^1^Department of Diagnostic Imaging, Polish Mothers' Memorial Hospital Research Institute, Łódź, Poland; ^2^Department of Neurosurgery and Neuro-Oncology, Barlicki University Hospital, Medical University of Lodz, Łódź, Poland; ^3^Pixel Technology, Łódź, Poland; ^4^Department of Anatomy, Aichi Medical University, Nagakute, Aichi, Japan; ^5^Department of Sleep Medicine and Metabolic Disorders, Medical University of Lodz, Łódź, Poland

**Keywords:** mass effect, automated segmentation, deep-learning neural network, intracranial compartments, cerebrospinal fluid reserve, traumatic brain injury

## Abstract

**Background:**

Intracranial space is divided into three compartments by the falx cerebri and tentorium cerebelli. We assessed whether cerebrospinal fluid (CSF) distribution evaluated by a specifically developed deep-learning neural network (DLNN) could assist in quantifying mass effect.

**Methods:**

Head trauma CT scans from a high-volume emergency department between 2018 and 2020 were retrospectively analyzed. Manual segmentations of intracranial compartments and CSF served as the ground truth to develop a DLNN model to automate the segmentation process. Dice Similarity Coefficient (DSC) was used to evaluate the segmentation performance. Supratentorial CSF Ratio was calculated by dividing the volume of CSF on the side with reduced CSF reserve by the volume of CSF on the opposite side.

**Results:**

Two hundred and seventy-four patients (mean age, 61 years ± 18.6) after traumatic brain injury (TBI) who had an emergency head CT scan were included. The average DSC for training and validation datasets were respectively: 0.782 and 0.765. Lower DSC were observed in the segmentation of CSF, respectively 0.589, 0.615, and 0.572 for the right supratentorial, left supratentorial, and infratentorial CSF regions in the training dataset, and slightly lower values in the validation dataset, respectively 0.567, 0.574, and 0.556. Twenty-two patients (8%) had midline shift exceeding 5 mm, and 24 (8.8%) presented with high/mixed density lesion exceeding >25 ml. Fifty-five patients (20.1%) exhibited mass effect requiring neurosurgical treatment. They had lower supratentorial CSF volume and lower Supratentorial CSF Ratio (both *p* < 0.001). A Supratentorial CSF Ratio below 60% had a sensitivity of 74.5% and specificity of 87.7% (AUC 0.88, 95%CI 0.82–0.94) in identifying patients that require neurosurgical treatment for mass effect. On the other hand, patients with CSF constituting 10–20% of the intracranial space, with 80–90% of CSF specifically in the supratentorial compartment, and whose Supratentorial CSF Ratio exceeded 80% had minimal risk.

**Conclusion:**

CSF distribution may be presented as quantifiable ratios that help to predict surgery in patients after TBI. Automated segmentation of intracranial compartments using the DLNN model demonstrates a potential of artificial intelligence in quantifying mass effect. Further validation of the described method is necessary to confirm its efficacy in triaging patients and identifying those who require neurosurgical treatment.

## 1 Introduction

The intracranial (IC) compartments, formed by the falx cerebri and tentorium cerebelli, have limited capacity to accommodate volume changes of the brain, blood, and cerebrospinal fluid (CSF) (Wilson, 2016). Brain injury results in reduction of CSF reserve that may lead to mass effect. This phenomenon can contribute to secondary injury, including cerebral edema, ischemia, and herniation. Further investigation is needed to understand the anatomical and pathological aspects of compartmental distribution of IC contents and its consequences.

Cerebrospinal fluid reserve is researched in terms of IC pressure (ICP) which is measured using intraventricular sensors, and volume which can be assessed on imaging studies (Dhar et al., 2021). However, there is no widely accepted method to quantify mass effect. The Marshall scale integrates qualitative aspects such as basal cistern effacement, midline shift exceeding 5 mm and high density lesion larger than 25 mm^3^, for prognostic assessment (Marshall et al., 1992). However, interrater variability may affect the results (Maas et al., 2005), and simplified formulas to ascertain the volumetric criterion may be imprecise (Vos et al., 2001). Automated volumetric evaluation may enhance its accuracy and assist in clinical decision-making at emergency departments without delays in diagnosis (Jain et al., 2019).

The growing demand for CT to detect IC hemorrhages and assess mass effect can be addressed through the use of artificial intelligence (AI) and machine learning (Chang et al., 2016; Heit et al., 2017; Raju et al., 2020; Brossard et al., 2021; Colasurdo et al., 2022). They are utilized in emergency care for various purposes, including triage, injury prediction, and outcome evaluation (Hunter et al., 2023). The ongoing efforts aim to automate lesion identification and segmentation, and assess CSF reserve (Monteiro et al., 2020; Colasurdo et al., 2022; Schmitt et al., 2022; Hunter et al., 2023; Yamada et al., 2023).

We conducted manual segmentation of IC compartments and threshold segmentation of CSF on emergency CT scans, which served as the ground truth. This data was then utilized as input for a deep-learning neural network (DLNN), which was trained to automate the segmentation task.

The main objective of this study was to develop an algorithm to quantify the mass effect requiring neurosurgical treatment on emergency head CT scans.

## 2 Methods

The study is in accordance with human rights declarations and regulations, and was approved by Institutional Review Board. Patient consent to the study was not required as it involved retrospective analysis of anonymized medical records. We screened head CT scans obtained from patients after traumatic brain injury (TBI) at a high-volume emergency department between 2018 and 2020. CT scans were performed on three scanners (Optima CT540, Revolution CT, Lightspeed VCT; GE Healthcare, USA). The manuscript was prepared following the CLAIM (Mongan et al., 2020) and the STROBE Guidelines.

### 2.1 CT screening and neurosurgical assessment

Studies with technical flaws, significant motion artifacts, or incomplete skull coverage were excluded. The presence of ischemia or hemorrhage, including subdural (SDH), epidural (EDH), intracerebral (ICH), cerebellar (CBH), subarachnoid (SAH), intraventricular (IVH), and contusions, was recorded. We undertook a thorough investigation to identify radiological criteria for mass effect necessitating neurosurgical treatment, drawing from the literature of the past two decades (Bullock et al., 2006a,b,c,d; Carney et al., 2017; Greenberg, 2019; Hawryluk et al., 2020; Greenberg et al., 2022). A summary of the criteria is shown in Supplementary Table S1. Neurosurgical assessment was independently carried out by three investigators, following the radiological criteria and clinical experience.

### 2.2 Manual segmentation

Two investigators segmented brain series of ≤1.25 mm slice thickness. The sagittal plane was manually adjusted to closely align with the falx cerebri, serving as the delineation between the left and right supratentorial compartments. During IC space segmentation we utilized the two-dimensional smart brush tool in Exhibeon3 DICOM viewer (Pixel Technology, Lodz, Poland) in the bone window (W: 2500 L: 800). The boundary with the spinal canal was drawn along the transverse plane, perpendicular to the established sagittal plane of the falx cerebri, intersecting the McRae line connecting *basion* and *opisthion* craniometric points. The boundaries with cranial openings were drawn in line with the inner surface of the cranium. The boundary between supra- and infratentorial compartments was delineated in the brain window (W: 80 L: 40) using multiplanar reconstructions, taking into account the course of the tentorium cerebelli. The tentorial notch was identified on coronal reconstructions as a line connecting the free edges of the tentorium cerebelli, and refined on transverse reconstructions. The three resulting compartments – right and left supratentorial and one infratentorial – covered everything inside the cranium, including the brain, CSF, and any potential pathologies. The sum of the volumes of the three compartments constitutes the IC space. Voxels exhibiting Hounsfield Unit (HU) values ranging from −5 to 15 were labeled as CSF. Presence of artifacts like beam hardening and pervasive noise, often led to misidentifying voxels as CSF, which was manually excluded.

### 2.3 Network architecture

We used a convolutional neural network with basic UNet architecture in a 3D version (Falk et al., 2019). The model takes in a single-channel image as input and produces seven channels of output with segmentation. The model’s encoder comprised five levels, with feature sizes of 32, 32, 64, 128, and 256, respectively. Leaky ReLU was employed as the activation layer (Xu et al., 2015). The total number of parameters in the model was 5.7 million. During the training process, the sum of Dice Loss and Cross Entropy was minimized using the AdamW optimizer. To schedule the learning rate, the One Cycle Scheduler technique was utilized with a maximum learning rate value of 0.001. PyTorch was used as a training framework. Augmentation and image processing was done in MonAI. Model weights were initialized randomly at the start of the training process.

### 2.4 Image preprocessing

**Figure 1:**
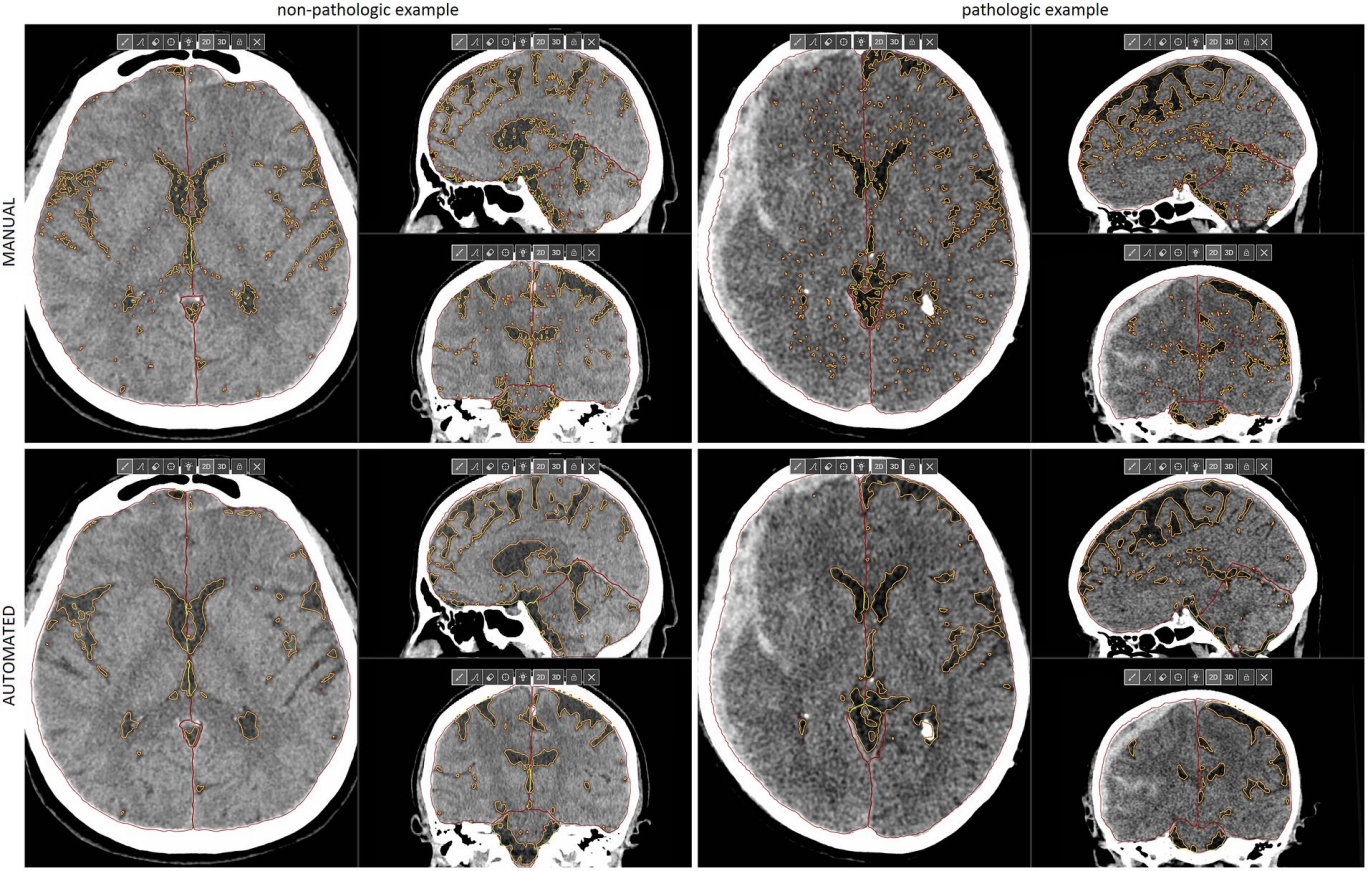
Multiplanar reconstructions of manual **(upper)** and automated **(lower)** segmentations of IC compartments and CSF in the non-pathologic **(left)** and pathologic example **(right)** of emergency CT scans. The latter example shows a right-sided acute SDH with significant mass effect that requires neurosurgical treatment, despite a relatively low midline shift. Reduced IC reserve in the right supratentorial compartment is well visualized. SDH, subdural hematoma, CSF, cerebrospinal fluid, CT, computed tomography, IC, intracranial. This figure is original to this submission so no credit or license is needed.

The training and validation datasets were randomly selected to ensure representative coverage of the entire available data (Table 1). We conducted several preprocessing steps before utilizing medical images as inputs for our model. Firstly, the images were resampled to a spacing of 1 millimeter to ensure consistency in resolution. Secondly, based on the Hounsfield Scale a threshold value of 100 was applied to retain only the most relevant information. Specifically, any pixel values above 100 were set to this value. Following this, the intensities of the remaining pixels were normalized to range between −1 and 1. To ensure the model was exposed to a diverse range of inputs during training, randomly selected preprocessed images were used with augmentations such as Gaussian Noise, random contrast adjustments, and rotations. This helped to train a robust model capable of handling varied inputs. Single voxels marked as CSF by the initial threshold, which might have corresponded to artifacts or small post-ischemic lesions, were excluded from CSF. This augmentation resulted in a more faithful representation of the ventricular system and subarachnoid reserve on the CT scans, aligning with human perception (Figure 1). Upon visual inspection, the final model, which exhibited the smallest variations in studies with the greatest ground truth discrepancies, was selected. The performance metrics of the optimal model across all data partitions are provided in Table 1 and remained similar and consistent across both the training (dependent) and validation (independent) datasets. Consequently, we used the DLNN predictions from both the training and validation datasets to evaluate the clinical efficacy of CSF Distribution Ratios.

**Table 1:** Patients characteristics and comparison of the DCS between training and validation datasets.

	Training dataset *n* = 189	Validation dataset *n* = 85
**Patients characteristics**		
Mean age	62 years ±18.7	59 years ±18.1
Acute SDH	64 (33.9%)	25 (29.4%)
Chronic SDH	27 (14.3%)	13 (15.3%)
EDH	9 (4.8%)	6 (7.1%)
ICH	53 (28%)	15 (17.6%)
CBH	5 (2.6%)	1 (1.2%)
Traumatic SAH	28 (14.8%)	4 (4.7%)
Spontaneous SAH	12 (6.3%)	3 (3.5%)
IVH	27 (14.3%)	7 (8.2%)
Contusions	63 (33.3%)	20 (23.5%)
Ischemia	60 (31.7%)	21 (24.7%)
Marshall classification		
- Diffuse injury I (no pathology) - Diffuse injury II - Diffuse injury III (swelling) - Diffuse injury IV (shift) - Evacuated mass lesion - Nonevacuated mass lesion	21 (11.1%) 113 (59.8%) 9 (4.8%) 1 (0.5%) 42 (22.2%) 3 (1.6%)	22 (25.9%) 45 (52.9%) 4 (4.7%) 0 13 (15.3%) 1 (1.2%)
**DSC**		
Average	0.782	0.765
Right supratentorial compartment	0.935	0.927
Left supratentorial compartment	0.932	0.927
Infratentorial compartment	0.905	0.903
Right supratentorial CSF	0.589	0.567
Left supratentorial CSF	0.615	0.574
Infratentorial CSF	0.572	0.556

The datasets were randomly selected to ensure representative coverage of the entire available data. CBH, cerebellar hemorrhage, CSF, cerebrospinal fluid, EDH, epidural hematoma, CT, computed tomography, DSC, Dice Similarity Coefficient, ICH, intracerebral hemorrhage, IVH, intraventricular hemorrhage, SAH, subarachnoid hemorrhage, SDH, subdural hematoma.

### 2.5 CSF distribution ratios

**Figure 2:**
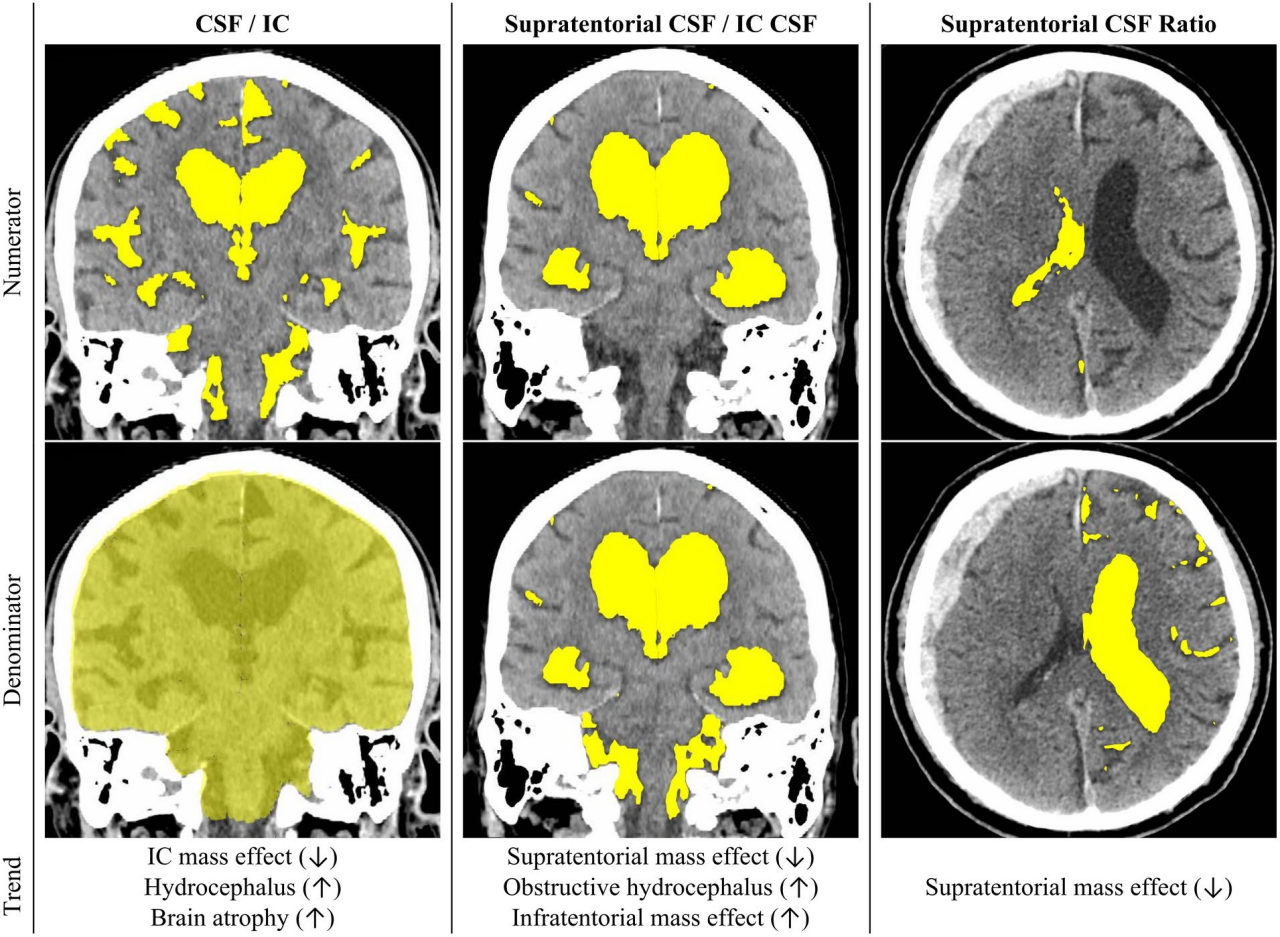
The illustration showcases the proposed CSF Distribution Ratios, with their definitions outlined in the manuscript. Rows one and two visualize the numerator and denominator, respectively, for each CSF Distribution Ratio. In row three, the figure delineates potential applications of each ratio and elucidates the directional changes associated with specific pathologies. CSF, cerebrospinal fluid, IC, intracranial, This figure is original to this submission so no credit or license is needed.

Volumetric data obtained from automated segmentation performed by the DLNN model was used to compute a series of quantitative indicators in each patient (Figure 2). The ratio “CSF/IC” refers to the proportion of CSF volume in relation to the IC space volume. The “Supratentorial CSF/IC CSF” represent the proportion of CSF in the supratentorial compartments relative to the CSF volume. The “Supratentorial CSF Ratio” quantifies the asymmetry in CSF distribution within the supratentorial compartments by dividing the volume of CSF on the side with reduced CSF reserve by the volume of CSF on the opposite side.

### 2.6 Statistical analysis

We used StatSoft Statistica (Tulsa, OK) and R Programming. Continuous variables were compared using Mann–Whitney *U* test. Categorical variables were compared using either Pearson’s chi-squared test or two-sided Fisher’s exact test. Predictive model was developed using logistic regression modelling with backward stepwise feature selection with likelihood ratio-test and with *p*-value of greater than 0.01 needed for stepwise feature removal. The heatmap was generated using unsupervised hierarchical clustering analysis with the *pheatmap* package in R Studio. Bland–Altman plots were generated using the ggplot2 package in R Studio. Power analysis for the test group was done using the pROC package. It yielded a required sample size of approximately 31 cases and 154 controls, with control-to-case ratio of 5, an anticipated area under the ROC curve of 0.7 and a desired power of 0.95 at a significance level of 0.05.

## 3 Results

**Figure 3:**
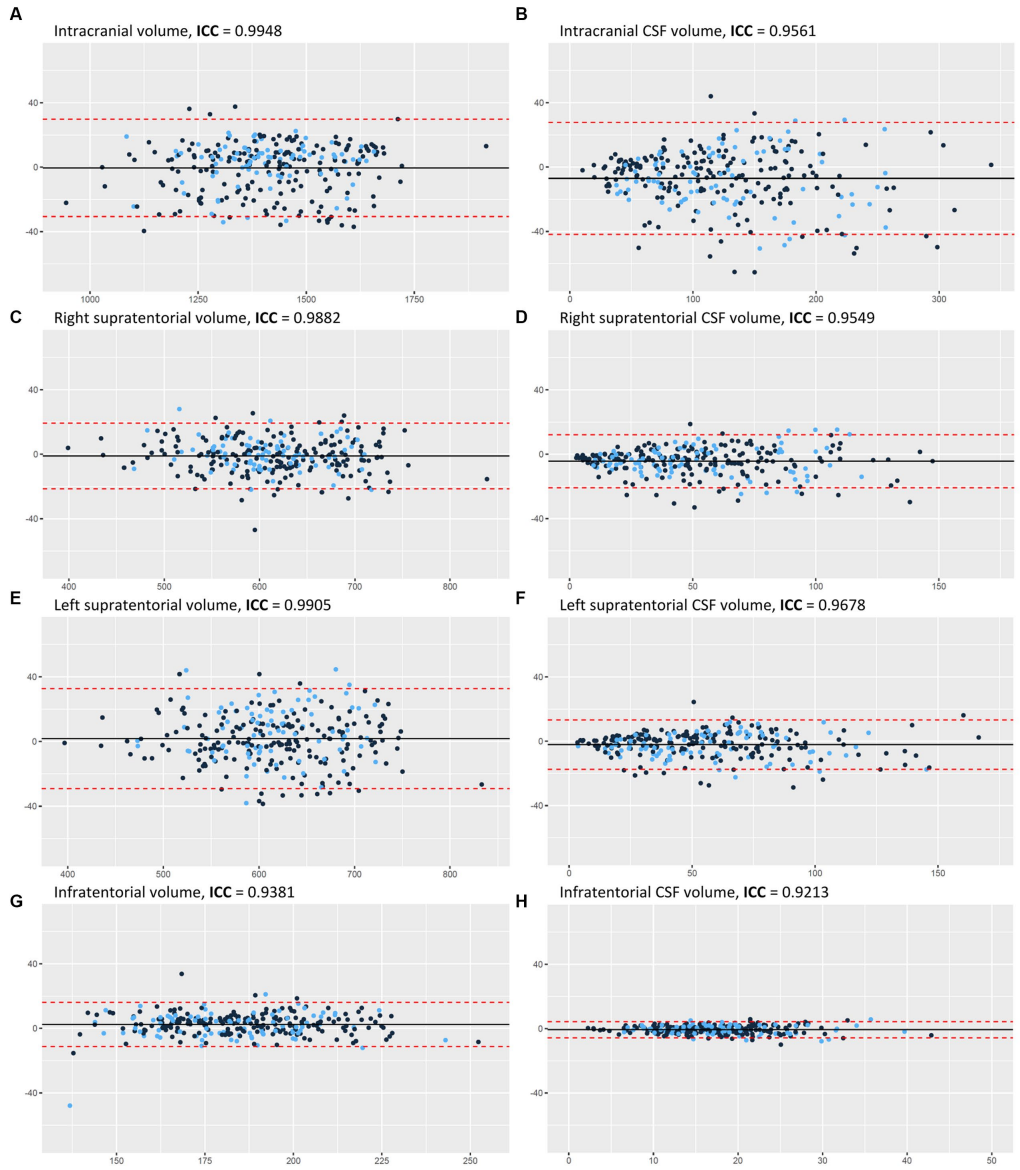
Bland–Altman plots for the manual and automated measurements of: **(A)** IC volume, **(B)** CSF volume, **(C)** right supratentorial volume, **(D)** right supratentorial CSF volume, **(E)** left supratentorial volume, **(F)** left supratentorial CSF volume, **(G)** infratentorial volume, and **(H)** infratentorial CSF volume. *Y* axes represent the difference between manual and automated measurements. *X* axes represent the average of manual and automated measurements. The color of each dot signifies the training (black) and the validation (blue) datasets. The black horizontal line indicates the mean measurement difference (bias), and if it is below zero it means that the average automated measurement was lower than the average manual measurement. The two red dashed horizontal lines represent the limit of agreement (1.96 × SD). AI, artificial intelligence, CSF, cerebrospinal fluid, IC, intracranial, ICC, intraclass correlation coefficient, SD, standard deviation. This figure is original to this submission so no credit or license is needed.

The study included 274 patients, mean age 61 years ±18.6. Example segmentations of the IC compartments and CSF are presented in Figure 1. The mean volumes are provided in Table 2. The intraclass correlation coefficient (ICC) values between manual and automated segmentations were all above 0.92 (Figure 3).

**Table 2:** Comparison of the volumes of IC compartments and CSF obtained from manual and automated segmentations of 274 head trauma CT scans performed at the emergency department.

Segmentation	Manual (ml)	Automated (ml)	ICC
Mean IC vol.	1415.9 ± 151	1416.3 ± 149.7	0.9948
Mean right supratentorial vol.	615 ± 68.3	613.9 ± 67.3	0.9882
Mean left supratentorial vol.	616.8 ± 67.9	615.8 ± 67.1	0.9905
Mean infratentorial vol.	184.1 ± 20.9	186.6 ± 21	0.9381
Mean CSF vol.	127.1 ± 65.2	120 ± 63	0.9561
Mean right supratentorial CSF vol.	52.9 ± 31.5	48.6 ± 31	0.9549
Mean left supratentorial CSF vol.	56.7 ± 32.1	54.6 ± 31.5	0.9678
Mean infratentorial CSF vol.	17.5 ± 6.7	16.8 ± 6.6	0.9213

CSF, cerebrospinal fluid, IC, intracranial, ICC, intraclass correlation coefficient, vol., volume.

Mass effect that required neurosurgical treatment was present in 55 patients (20.1%). Supratentorial CSF Ratio below 60% demonstrated a sensitivity of 74.5% and specificity of 87.7% in accurately identifying these patients. The ROC curve illustrated an AUC of 0.88 (Supplementary Figure S1). Noteworthy, neurosurgery for mass effect was never indicated in patients whose CSF constituted 10–20% of the IC space, with 80–90% being supratentorial, and whose Supratentorial CSF Ratio was larger than 80%. Uni- and multivariate analyses of radiological predictors of mass effect requiring neurosurgical treatment is provided in Table 3. Based on the selected CSF Distribution Ratios, we created a triage protocol for patients at the emergency department (Table 4).

**Table 3:** Radiological predictors of mass effect requiring neurosurgical treatment in 274 patients who were diagnosed at the emergency department.

	All	Neurosurgical treatment	Univariate OR (95% CI)	Multivariate OR (95% CI)	*value of p*
Total	274	55 (20.1%)			
Bilateral Lesions	81	25 (30.9%)	2.4 (1.3–4.5)	–	
Acute SDH	89	21 (23.6%)	1.4 (0.7–2.5)	–	
Chronic SDH	40	19 (47.5%)	4.9 (2.4–10.2)	15.1 (3.9–58.9)	<0.001
EDH	15	7 (46.7%)	3.8 (1.3–11.1)	–	
ICH	68	27 (39.7%)	4.2 (2.2–7.9)	8.0 (2.3–28.1)	0.001
CBH	6	3 (50.0%)	4.2 (0.8–21.2)	–	
Contusions	83	15 (18.1%)	0.8 (0.4–1.6)	–	
Traumatic SAH	32	6 (18.8%)	0.9 (0.3–2.3)	–	
Spontaneous SAH	15	4 (26.7%)	1.5 (0.5–4.9)	–	
IVH	34	13 (38.2%)	2.9 (1.4–6.3)	–	
Ischemia	82	19 (23.2%)	1.3 (0.7–2.5)	–	
Basal Cisterns Compressed	41	25 (61.0%)	10.6 (5.1–22.1)	–	
Basal Cisterns Absent	11	10 (90.9%)	48.4 (6.0–388)	388 (24.7–6,111)	<0.001
MLS > 5 mm	22	21 (95.5%)	134 (17.5–1,033)	–	
High/Mixed Density Lesion>25 mL	24	20 (83.3%)	30.7 (9.9–95.2)	14 (2.9–67)	<0.001
Supratentorial CSF Ratio (continuous variable)	81% (62–92%)	40% (28–65%)	>999	1,072 (87.1–13,221)	<0.001
Supratentorial CSF Ratio < 60%	68	41 (60.3%)	20.8 (10.1–43.1)	–	
Supratentorial CSF Ratio > 80%	147	6 (4.1%)	0.07 (0.03–0.17)	–	
Supratentorial CSF/IC CSF (continuous variable)	86% (80–89%)	81% (70–87%)	758 (33.1–17,363)	–	
Supratentorial CSF/IC CSF 80–90%	153	25 (16.3%)	0.59 (0.33–1.07)	–	
IC CSF/IC volume (continuous variable)	8% (5–11%)	6% (3–9%)	>999	-	
IC CSF/IC volume 10–20%	94	9 (9.6%)	0.31 (0.14–0.66)	-	

CSF Distribution Ratios were calculated using automated segmentation by the DLNN model developed specifically for this study. Continuous variables are presented as medians and IQR. Supratentorial CSF Ratio was defined as a ratio of ipsilateral and contralateral supratentorial CSF volumes. “Ipsilateral” and “contralateral” refer to the supratentorial compartment with reduced CSF reserve. CBH, cerebellar hemorrhage; CSF, cerebrospinal fluid; EDH, epidural hemorrhage; IC, intracranial; ICH, intracerebral hemorrhage; IQR, interquartile range; IVH, intraventricular hemorrhage; MLS, midline shift; NS, not significant; SAH, subarachnoid hemorrhage; SDH, subdural hemorrhage.

**Table 4:** Triage protocol for mass effect that requires neurosurgical treatment based on the three selected CSF Distribution Ratios obtained from automated segmentation using the DLNN model developed specifically for this study.

**Triage**	**Criteria**	**Mass effect requiring neurosurgical treatment**
**Red** *Immediate*	Supratentorial CSF Ratio < 60%	41/68 (60%)
**Yellow** *Urgent*	*other patients*	14/168 (8%)
**Green** *Low risk*	Supratentorial CSF Ratio > 80% *and* CSF**/** IC vol. 10–20% *and* Supratentorial CSF**/** IC CSF 80–90%	0/38

CSF, cerebrospinal fluid, DLNN, deep-learning neural network, IC, intracranial, vol., volume.

**Figure 4:**
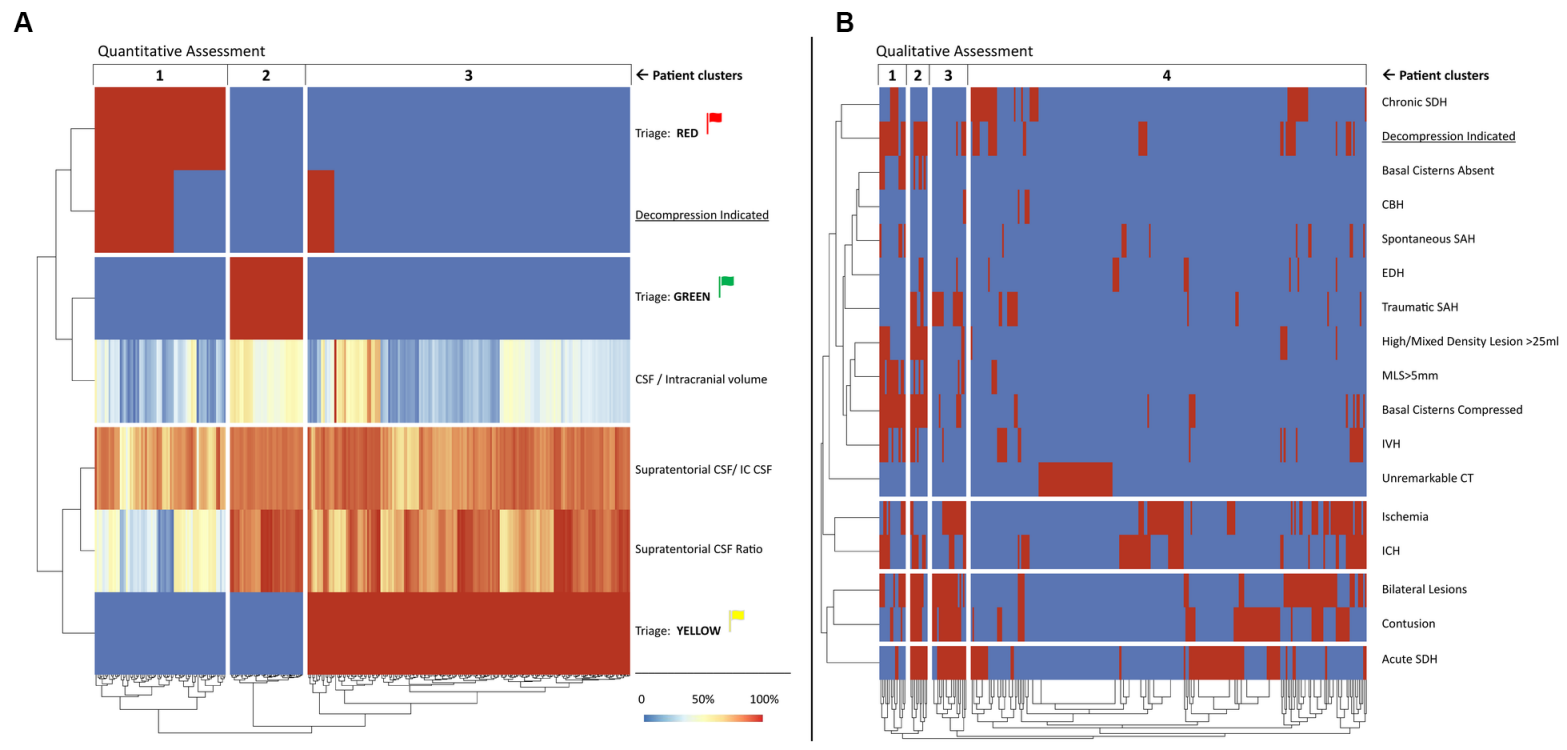
Heatmap representation of unsupervised HCA of selected quantitative **(A)** and qualitative **(B)** predictors of mass effect that requires neurosurgical treatment in patients after emergency head CT scans. Each column represents one patient, and they are grouped into clusters according to unsupervised HCA. The quantitative assessment **(A)** shows the selected CSF Distribution Ratios calculated from automated segmentation of IC compartments and CSF volumes and the proposed triage system presented in the Table 4, whereas the qualitative assessment **(B)** was based on the radiological reports and simplified formulas used to ascertain the volumetric criterion. Red indicates “yes” and blue indicates “no.” Color legend for the continuous variables is provided in the diagram. Explanation and interpretation of the findings depicted in the figure can be found in the Results section of the article. CSF, cerebrospinal fluid leak, CT, computed tomography, HCA, hierarchical clustering analysis, IC, intracranial. This figure is original to this submission so no credit or license is needed.

By utilizing unsupervised hierarchical clustering analysis (HCA), patients (columns) were grouped according to A the triage protocol based on the selected CSF Distribution Ratios (Figure 4A) the presence and type of IC bleeding, any high or mixed density lesion larger than 25 mL, midline shift greater than 5 mm, and appearance of basal cisterns (Figure 4B).

Quantitative assessment (Figure 4A) associated with the triage protocol revealed three clusters of patients. The first cluster contained patients marked in red according to triage protocol, among whom 41 (60.3%) required neurosurgical treatment. In this group, all patients had a Supratentorial CSF Ratio below 60%. The second cluster contained patients marked in green who did not require neurosurgical treatment. All showed a balanced CSF distribution between IC compartments, and a Supratentorial CSF Ratio close to 1. The third cluster contains patients marked in yellow, among whom 14 (8%) required neurosurgical treatment. This is the largest and most heterogeneous group.

HCA based on the qualitative assessment is provided in Figure 4B. Clusters one and two were composed of patients with compressed basal cisterns, most of whom required surgery, whereas, patients in cluster three usually did not require surgery and were characterized by bilateral lesions, contusions, ischemia, traumatic SAH, and acute SDH. Cluster four included more than three-quarters of patients with either unremarkable head CT or surgical indications due to various lesions. HCA analysis highlights that incorporating the triage protocol based on the selected CSF Distribution Ratios could improve the accuracy of determining the need for neurosurgical treatment.

## 4 Discussion

**Figure 5:**
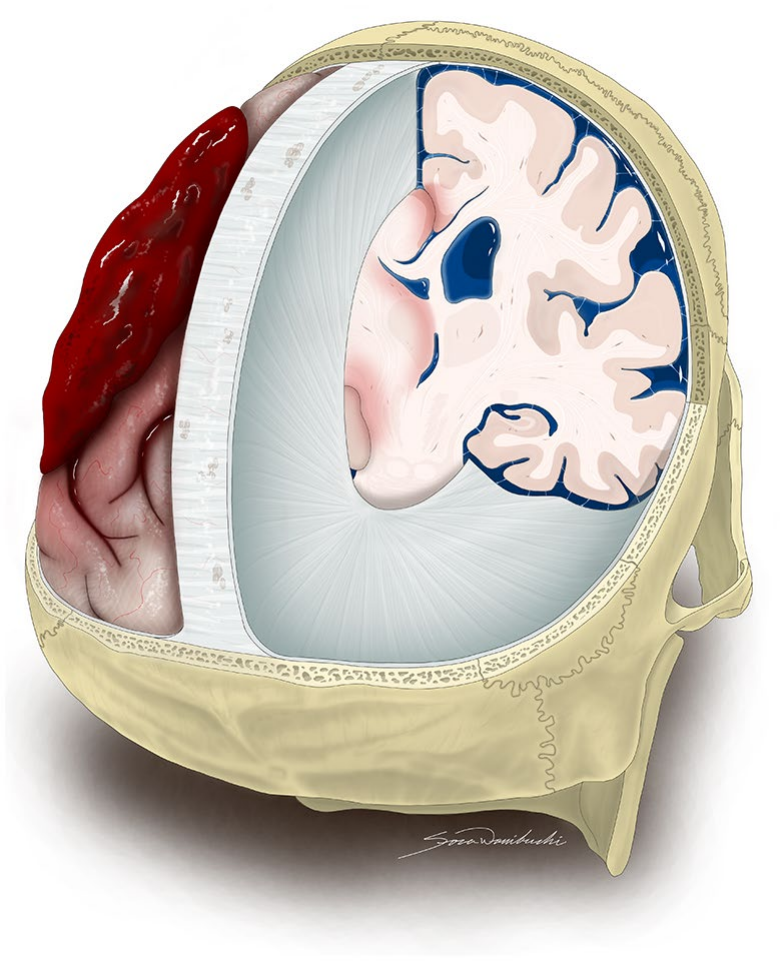
An artistic representation of oblique posterior view of IC contents revealing mass effect that requires neurosurgical treatment due to a left-sided acute SDH. The illustration exhibits the removal of both sides of the skull, as well as the dura mater covering the right and left hemispheres, except for the regions of the falx cerebri and tentorium cerebelli in order to show the limited capacity of the IC compartments to accommodate volume changes of the brain, blood, and CSF. The acute SDH over the left cerebral hemisphere causes midline shift, while the CSF reserve remains unaffected on the right side. SDH, subdural hematoma, CSF, cerebrospinal fluid leak, IC, intracranial. This figure is original to this submission so no credit or license is needed.

Automated segmentation of IC compartments and CSF might contribute to fast, accurate, and consistent diagnosis of neurological emergencies. The underlying hypothesis is that various pathologies that require neurosurgical treatment, such as hemorrhage, brain edema, hydrocephalus or infarction, present as a mass effect associated with CSF displacement (Chen et al., 2016; Bobeff et al., 2018; Mönch et al., 2020; Dhar et al., 2021) (Figure 5). Our key findings are: (1) there was strong agreement between manual and automated segmentations of IC compartments and CSF that support further validation of the latter and its use in clinical scenario, (2) CSF Distribution Ratios may help quantify mass effect and improve radiological reports without increasing time burdens.

Evaluation of automated segmentations was done in the context of Dice Similarity Coefficient (DSC), volumetric measurements, and through an unmediated evaluation of images with a focus on the most outliers. DSC for training and validation datasets were broadly equivalent (Table 1) and ICC very high (Table 2); furthermore, upon visual assessment, automated segmentation excelled in accurately identifying CSF and effectively partitioning IC compartments (Figure 1).

Emergency CT imaging aims to identify primary injuries, such as extraaxial hematomas, cerebral hemorrhage, contusion, and skull fractures. It also assesses their impact on IC contents, resulting in cerebral edema and increased ICP (Rincon et al., 2016). Both primary and secondary injuries reduce CSF reserve in the affected IC compartment or reduce the overall IC reserve in case of diffuse injury. In fact, radiological manifestations such as sulcal marking obliteration and brain displacement into sulci, cisterns, and ventricles, can be more challenging to observe than primary injuries itself.

Quantifying mass effect can improve the interpretation of radiological findings and reduce reliance on subjective descriptions with variable agreement among raters. Currently, there is no standardized method for quantitatively assessing mass effect, apart from midline shift. The evaluation of radiological findings indicating increased ICP relies on the expertise of neurosurgeons and radiologists. Common terms used in radiological reports include “CSF reserve reduction/loss,” “sulci effacement/loss,” accompanied by specifying the location, such as “right-sided supratentorial” or “infratentorial.” They are primarily qualitative and may not convey precise information. Our results show that CSF Distribution Ratios offer a valuable and potentially reproducible method to quantify mass effect.

Triaging imaging studies becomes increasingly important with the spread of teleradiology that potentially leads to delays in diagnosis. The use of CSF Distribution Ratios can prioritize cases with the utmost urgency, expedite radiology reports, and facilitate consultation between clinicians and radiologists, especially in centers with large numbers of CT scans (O'Neill et al., 2020). Possible triage criteria for categorizing patients into 3 risk groups of mass effect are outlined in Table 4. To comprehensively represent IC conditions, the protocol includes prognostic factors validated in univariate analysis and describes CSF reserve, supratentorial CSF asymmetry, and infra- and supratentorial CSF distribution.

Remote neurosurgical consultations frequently take place in distant hospitals and, if patient transportation is required, entail substantial costs and time. Frequently conservative therapy is preferred, still the stigma associated with IC hemorrhage, even without the need for neurosurgical treatment, can result in unnecessary patient transport. Automated segmentation and quantitative evaluation offer a precise and timely approach. This approach can be critical in situations where patient transport is risky and immediate surgery is being considered. It could facilitate remote neurosurgical consultations and aid in early-stage diagnosis at the emergency department.

The ratios “CSF/IC” and “supratentorial CSF/IC CSF” may capture nuances in mass effect resulting from infratentorial lesions and hydrocephalus due to *aqueductal stenosis*. The diagnosis of hydrocephalus requires clinical expertise and careful evaluation of signs and symptoms. CSF Distribution Ratios could enable more precise assessment of subsequent examinations within the same patient to achieve a more accurate and comprehensive disease monitoring.

Other pathologies that should be considered during the assessment of CSF reserve, where no localized primary injury is evident, include inflammatory or infectious processes, demyelinating diseases, vascular malformations, and metabolic disorders. Unless there is previous CT, it is often difficult to judge whether CSF reserve is within normal limits, diminished or severely diminished as a result of edema and mild brain swelling. Percentile grids for IC contents normalized by IC volume, gender, and age could guide radiologists by highlighting values outside established thresholds. For example, if normalized CSF reserve is below 3rd percentile, general brain swelling could be considered in impressions of radiologic report. Percentile grids could also help in cases of brain atrophy, a natural phenomenon associated with aging but not directly measured in clinical practice. In cases of cerebral atrophy, there is a notable reduction in the volume of both white and grey matter, which is subsequently supplanted by CSF. This phenomenon manifests radiologically as an enlargement of the lateral ventricles and widening of the arachnoid space fissures. Consequently, volumetric assessments reveal an increased CSF volume, leading to an increased CSF / IC ratio. Hence, the extent of cerebral atrophy can be quantitatively evaluated through our method, which leverages these radiological and volumetric changes.

### 4.1 Limitations

It was a single center study. We acknowledge the heterogeneity of our patient cohort, consisting of individuals who experienced TBI and were diagnosed at the emergency department. On one hand this contributed to the diversity of mass effect presentations, including CBH, SDH, global edema, and hydrocephalus, on the other highlighted the method’s versatility as the precise cutoff points could be tailored in specific pathologies. Another limitation of our study is the absence of detailed information on which patients with hydrocephalus required drainage procedures, limiting our ability to robustly assess the effectiveness of the presented ratios in predicting the need for such interventions. Our sample had a small number of infratentorial lesions. Reproducibility of our DLNN model was not subject to test–retest assessment. HCA of quantitative variables and one qualitative variable is very likely to split the group based on the latter; however, our goal was to show correlations between CSF Distribution Ratios and mass effect requiring neurosurgical treatment. We did not consider clinical factors related to patients condition that may influence the decision to perform neurosurgery, such as patient age, functional status, Glasgow Coma Scale (GCS) score, and comorbidities; however, this was our assumption that the model should identify radiological predictors, and the final treatment decision is made by clinicians, who take into consideration all available information. The role of the DLNN is to provide accurate and timely information, but not to replace a trained neuroradiologist. Going forward, we plan to integrate lesion volume calculations into our algorithm to enhance its capabilities and provide precise cutoff points for particular lesions.

## 5 Conclusion

Automated segmentation of IC compartments and calculation of CSF Distribution Ratios may enhance clinical decision-making and improve emergency management. The DLNN model effectively partitions the IC space into supra- and infratentorial compartments. CSF Distribution Ratios offer timely estimation of CSF reserve thus may enhance the predictive value of radiological reports. The integration of AI into the medical field can enhance the accuracy and speed of clinical diagnosis. Further research and implementation of AI into the healthcare system present an area of great interest bearing in mind their promising potential.

## Data availability statement

The datasets presented in this article are not readily available because the dataset consisted only of computed tomography images. Requests to access the datasets should be directed to m.grobelna@pixel.com.pl.

## Ethics statement

The studies involving humans were approved by Institutional Review Board approval RNN/211/16/KE. The studies were conducted in accordance with the local legislation and institutional requirements. The ethics committee/institutional review board waived the requirement of written informed consent for participation from the participants or the participants’ legal guardians/next of kin because there was a retrospective study design.

## Author contributions

TP: Conceptualization, Data curation, Formal analysis, Funding acquisition, Investigation, Methodology, Project administration, Resources, Software, Writing – original draft. KM: Data curation, Formal analysis, Writing – original draft. KW: Formal analysis, Investigation, Writing – original draft. MG: Investigation, Software, Writing – original draft. SW: Visualization, Writing – original draft. DJ: Methodology, Supervision, Writing – review & editing. EB: Conceptualization, Data curation, Formal analysis, Funding acquisition, Investigation, Methodology, Project administration, Resources, Software, Supervision, Validation, Visualization, Writing – original draft, Writing – review & editing.

## References

[ref1] Bobeff E. J. Fortuniak J. Bobeff K. Ł. Wiśniewski K. Wójcik R. Stefańczyk L.. (2018). Diagnostic value of lateral ventricle ratio: a retrospective case-control study of 112 acute subdural hematomas after non-severe traumatic brain injury. Brain Inj. 33, 226–232. doi: 10.1080/02699052.2018.1539871 30417687

[ref2] Brossard C. Lemasson B. Attyé A. de Busschère J. A. Payen J. F. Barbier E. L.. (2021). Contribution of CT-scan analysis by artificial intelligence to the clinical care of TBI patients. Front. Neurol. 12:666875. doi: 10.3389/fneur.2021.666875, PMID: 34177773 PMC8222716

[ref3] Bullock M. R. Chesnut R. Ghajar J. Gordon D. Hartl R. Newell D. W.. (2006a). Surgical management of acute epidural hematomas. Neurosurgery 58, S2-7–S2-15. doi: 10.1227/01.NEU.0000210363.91172.A8, PMID: 16710967

[ref4] Bullock M. R. Chesnut R. Ghajar J. Gordon D. Hartl R. Newell D. W.. (2006c). Surgical management of posterior fossa mass lesions. Neurosurgery 58, S2-47–S2-55. doi: 10.1227/01.NEU.0000210366.36914.38 16540745

[ref5] Bullock M. R. Chesnut R. Ghajar J. Gordon D. Hartl R. Newell D. W.. (2006d). Surgical management of traumatic parenchymal lesions. Neurosurgery 58, S2-25–S2-46. doi: 10.1227/01.NEU.0000210365.36914.E3 16540746

[ref6] Bullock M. R. Chesnut R. Ghajar J. Gordon D. Hartl R. Newell D. W.. (2006b). Surgical management of acute subdural hematomas. Neurosurgery 58, S16–S24. PMID: 16710968

[ref7] Carney N. Totten A. M. O'Reilly C. Ullman J. S. Hawryluk G. W. J. Bell M. J.. (2017). Guidelines for the management of severe traumatic brain injury. Neurosurgery 80, 6–15. doi: 10.1227/NEU.0000000000001432, PMID: 27654000

[ref8] Chang J. C. Lin Y. Y. Hsu T. F. Chen Y. C. How C. K. Huang M. S. (2016). Trends in computed tomography utilisation in the emergency department: a 5 year experience in an urban medical Centre in northern Taiwan. Emerg. Med. Australas. 28, 153–158. doi: 10.1111/1742-6723.12557, PMID: 26991856

[ref9] Chen Y. Dhar R. Heitsch L. Ford A. Fernandez-Cadenas I. Carrera C.. (2016). Automated quantification of cerebral edema following hemispheric infarction: application of a machine-learning algorithm to evaluate CSF shifts on serial head CTs. Neuroimage Clin. 12, 673–680. doi: 10.1016/j.nicl.2016.09.018, PMID: 27761398 PMC5065050

[ref10] Colasurdo M. Leibushor N. Robledo A. Vasandani V. Luna Z. A. Rao A. S.. (2022). Automated detection and analysis of subdural hematomas using a machine learning algorithm. J. Neurosurg. 138, 1–8. doi: 10.3171/2022.8.JNS22888, PMID: 36461839

[ref11] Dhar R. Hamzehloo A. Kumar A. Chen Y. He J. Heitsch L.. (2021). Hemispheric CSF volume ratio quantifies progression and severity of cerebral edema after acute hemispheric stroke. J. Cereb. Blood Flow Metab. 41, 2907–2915. doi: 10.1177/0271678X211018210, PMID: 34013805 PMC8756467

[ref12] Falk T. Mai D. Bensch R. Çiçek Ö. Abdulkadir A. Marrakchi Y.. (2019). U-net: deep learning for cell counting, detection, and morphometry. Nat. Methods 16, 67–70. doi: 10.1038/s41592-018-0261-2 30559429

[ref13] Greenberg M. S. (2019). Handbook of neurosurgery. 9th Edn, New York: Thieme Medical Publishers.

[ref14] Greenberg S. M. Ziai W. C. Cordonnier C. Dowlatshahi D. Francis B. Goldstein J. N.. (2022). 2022 guideline for the Management of Patients with Spontaneous Intracerebral Hemorrhage: a guideline from the American Heart Association/American Stroke Association. Stroke 53, e282–e361. doi: 10.1161/STR.0000000000000407, PMID: 35579034

[ref15] Hawryluk G. W. J. Rubiano A. M. Totten A. M. O’Reilly C. Ullman J. S. Bratton S. L.. (2020). Guidelines for the Management of Severe Traumatic Brain Injury: 2020 update of the decompressive Craniectomy recommendations. Neurosurgery 87, 427–434. doi: 10.1093/neuros/nyaa278, PMID: 32761068 PMC7426189

[ref16] Heit J. J. Iv M. Wintermark M. (2017). Imaging of intracranial hemorrhage. J Stroke 19, 11–27. doi: 10.5853/jos.2016.00563 28030895 PMC5307932

[ref17] Hunter O. F. Perry F. Salehi M. Bandurski H. Hubbard A. Ball C. G.. (2023). Science fiction or clinical reality: a review of the applications of artificial intelligence along the continuum of trauma care. World J. Emerg. Surg. 18:16. doi: 10.1186/s13017-022-00469-1, PMID: 36879293 PMC9987401

[ref18] Jain S. Vyvere T. V. Terzopoulos V. Sima D. M. Roura E. Maas A.. (2019). Automatic quantification of computed tomography features in acute traumatic brain injury. J. Neurotrauma 36, 1794–1803. doi: 10.1089/neu.2018.6183, PMID: 30648469 PMC6551991

[ref19] Maas A. I. Hukkelhoven C. W. Marshall L. F. Steyerberg E. W. (2005). Prediction of outcome in traumatic brain injury with computed tomographic characteristics: a comparison between the computed tomographic classification and combinations of computed tomographic predictors. Neurosurgery 57, 1173–1182. doi: 10.1227/01.neu.0000186013.63046.6b, PMID: 16331165

[ref20] Marshall L. F. Marshall S. B. Klauber M. R. Van Berkum Clark M. Eisenberg H. Jane J. A.. (1992). The diagnosis of head injury requires a classification based on computed axial tomography. J. Neurotrauma 9, S287–S292. 1588618

[ref21] Mönch S. Sepp D. Hedderich D. Boeckh-Behrens T. Berndt M. Maegerlein C.. (2020). Impact of brain volume and intracranial cerebrospinal fluid volume on the clinical outcome in endovascularly treated stroke patients. J. Stroke Cerebrovasc. Dis. 29:104831. doi: 10.1016/j.jstrokecerebrovasdis.2020.104831, PMID: 32404285

[ref22] Mongan J. Moy L. Kahn C. E. Jr. (2020). Checklist for artificial intelligence in medical imaging (CLAIM): a guide for authors and reviewers. Radiol. Artif. Intell. 2:e200029. doi: 10.1148/ryai.2020200029, PMID: 33937821 PMC8017414

[ref23] Monteiro M. Newcombe V. F. J. Mathieu F. Adatia K. Kamnitsas K. Ferrante E.. (2020). Multiclass semantic segmentation and quantification of traumatic brain injury lesions on head CT using deep learning: an algorithm development and multicentre validation study. Lancet Digit Health. 2, e314–e322. doi: 10.1016/S2589-7500(20)30085-6, PMID: 33328125

[ref24] O'Neill T. J. Xi Y. Stehel E. Browning T. Ng Y. S. Baker C.. (2020). Active reprioritization of the Reading worklist using artificial intelligence has a beneficial effect on the turnaround time for interpretation of head CT with intracranial hemorrhage. Radiol. Artif. Intell. 3:e200024. doi: 10.1148/ryai.2020200024, PMID: 33937858 PMC8043365

[ref25] Raju B. Jumah F. Ashraf O. Narayan V. Gupta G. Sun H.. (2020). Big data, machine learning, and artificial intelligence: a field guide for neurosurgeons [published online ahead of print, 2020 Oct 2]. J. Neurosurg. 135, 1–11. doi: 10.3171/2020.5.JNS201288, PMID: 33007750

[ref26] Rincon S. Gupta R. Ptak T. (2016). “Imaging of head trauma” in Neuroimaging part I. Handbook of clinical neurology, *vol. 135* Eds. J. C. Masdeu and R. Gilberto González (Elsevier) 447–477. 27432678 10.1016/B978-0-444-53485-9.00022-2

[ref27] Schmitt N. Mokli Y. Weyland C. S. Gerry S. Herweh C. Ringleb P. A.. (2022). Automated detection and segmentation of intracranial hemorrhage suspect hyperdensities in non-contrast-enhanced CT scans of acute stroke patients. Eur. Radiol. 32, 2246–2254. doi: 10.1007/s00330-021-08352-4, PMID: 34773465 PMC8921016

[ref28] Vos P. E. van Voskuilen A. C. Beems T. Krabbe P. F. Vogels O. J. (2001). Evaluation of the traumatic coma data bank computed tomography classification for severe head injury. J. Neurotrauma 18, 649–655. doi: 10.1089/089771501750357591, PMID: 11497091

[ref29] Wilson M. H. (2016). Monro-Kellie 2.0: the dynamic vascular and venous pathophysiological components of intracranial pressure. J. Cereb. Blood Flow Metab. 36, 1338–1350. doi: 10.1177/0271678X16648711, PMID: 27174995 PMC4971608

[ref30] Xu B. Naiyan W. Chen T. Li M. (2015). Empirical evaluation of rectified activations in convolutional network. *ArXiv.org*. 1505.00853. doi: 10.48550/arXiv.1505.00853,

[ref31] Yamada S. Otani T. Ii S. Kawano H. Nozaki K. Wada S.. (2023). Aging-related volume changes in the brain and cerebrospinal fluid using artificial intelligence-automated segmentation [published online ahead of print, 2023 Apr 15]. Eur. Radiol. 33, 7099–7112. doi: 10.1007/s00330-023-09632-x, PMID: 37060450 PMC10511609

