# Supplementary material for: Automated volumetric evaluation of intracranial compartments and cerebrospinal fluid distribution on emergency trauma head CT scans to quantify mass effect
Source: Front Neurosci. 2024 Feb 19;18:1341734. doi: 10.3389/fnins.2024.1341734 (PMC10913188; doi:10.3389/fnins.2024.1341734)
Supplement: Supplementary file 1 [file Table_1.docx]

|  | **Radiological criteria for  mass effect requiring neurosurgical treatment**^17-24^ |
| --- | --- |
| **SDH** | - thickness greater than 10 mm - midline shift greater than 5 mm - herniation |
| **EDH** | - volume >30 mL - thickness greater than 15 mm - midline shift greater than 5 mm - herniation |
| **ICH** | - volume >10 ml to 30 mL - midline shift greater than 5 mm - herniation |
| **CBH** | - volume >15 mL - diameter > 30 mm - obstructive hydrocephalus - brainstem compression - herniation |
| **IVH** | - obstructive hydrocephalus - herniation |
| **Contusion** | - temporal contusions >20 mL - midline shift greater than 5 mm - herniation |
| **Brain edema** | - midline shift greater than 5 mm - herniation |

**Supplementary Table 1**. Radiological criteria for mass effect that requires neurosurgical treatment based on established guidelines. Abbreviations: CBH, cerebellar hematoma, EDH, epidural hematoma, ICH, intracerebral hemorrhage, IVH, intraventricular hemorrhage, SDH, subdural hematoma.
