# Supplementary figures and images for: Automated volumetric evaluation of intracranial compartments and cerebrospinal fluid distribution on emergency trauma head CT scans to quantify mass effect
Source: Front Neurosci. 2024 Feb 19;18:1341734. doi: 10.3389/fnins.2024.1341734 (PMC10913188; doi:10.3389/fnins.2024.1341734)

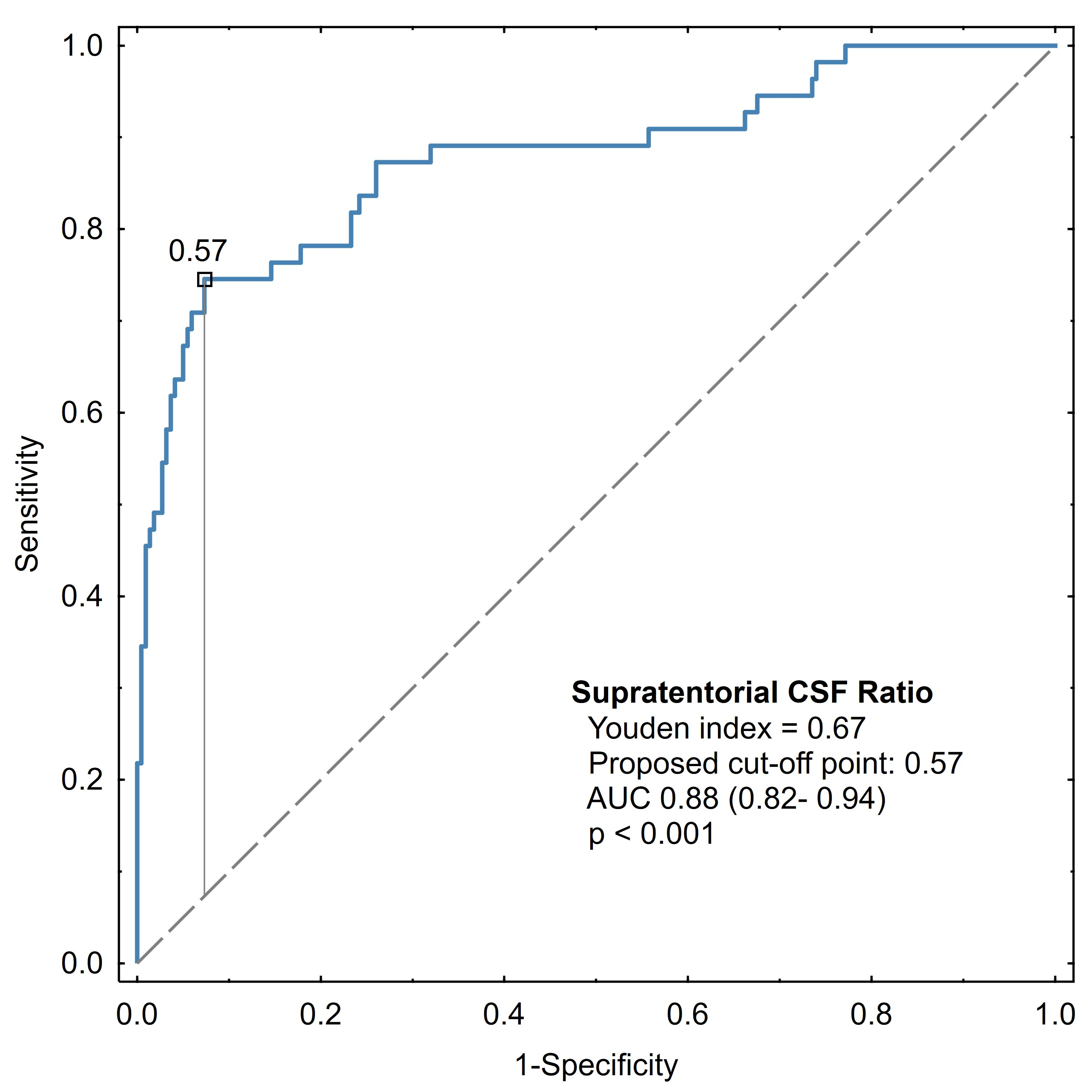

Supplement: Supplementary file 2 [file Image_1.TIF]
